# A Review of the Expansion and Integration of Production Line Balancing Problems: From Core Issues to System Integration

**DOI:** 10.3390/s25206337

**Published:** 2025-10-14

**Authors:** Adilanmu Sitahong, Zheng Lu, Yiping Yuan, Peiyin Mo, Junyan Ma

**Affiliations:** 1 School of Mechanical Engineering, Xinjiang University, Urumqi 830047, China; 19171160669@163.com (Y.Y.); peiyinmo@163.com (P.M.); 2 College of Civil Engineering and Architecture, Xinjiang University, Urumqi 830047, China; luzheng@stu.xju.edu.cn; 3 School of Energy Engineering, Xinjiang Engineering University, Urumqi 830023, China; 18154310990@163.com

**Keywords:** line balancing, smart manufacturing, human-robot collaboration, digital twin, design for manufacturing

## Abstract

The Line Balancing Problem (LBP) is a classic optimization topic in production management, aiming to improve efficiency through task allocation. With the transformation of the manufacturing industry towards intelligence, customization, and sustainability, its research scope has been significantly expanded. This study systematically reviews the recent research progress and proposes the C|H|V|E framework to analyze the LBP in four dimensions: (i) extension of the core line problem; (ii) horizontal integration with shop-floor decision-making; (iii) vertical coordination with enterprise-level operations; and (iv) extension of the value from efficiency improvement to sustainability and resilience enhancement. The review focuses on emerging trends, including artificial intelligence and data-driven approaches, digital twin-based optimization, flexible human-machine collaboration, and system integration across the lifecycle and circular economy. This paper provides a systematic overview of the current state of LBP research and explains how it continues to expand its boundaries by incorporating knowledge from new fields.

## 1. Introduction

The manufacturing industry is facing unprecedented challenges due to globalization, competition, and the demand for personalized manufacturing. As the core of the manufacturing industry, the operational efficiency, cost control, and rapid response to the market of production lines directly determine the core competitiveness of enterprises. Production line balancing, as a key technology, is widely used in production line optimization [1,2,3,4]. This technology aims to maximize resource utilization and meet production cycle requirements by reasonably allocating production tasks to each workstation [5,6,7], thereby enabling the assembly line to achieve smooth and efficient production [8,9].

The Simple Assembly Line Balancing Problem (SALBP) serves as the foundation for research in this field [10]. SALBP is defined as comprising SALBP-I and SALBP-II [11], representing the minimization of workstation count (Type I) and cycle time (Type II) under given production cycle and task sequence constraints [12,13]. The problem has established a solid foundation for research in the field of production line balancing [14].

However, due to the continuous development of today’s manufacturing industry, LBPs have become more complex, and their study has long gone beyond the traditional scope. On the one hand, manufacturing companies have to solve inherent complex problems, including mixed production, nonlinear (U-shaped, two-sided, etc.) layouts, worker heterogeneity, equipment failures, and uncertainty in task times [15]. On the other hand, the production line is no longer a unit that can be optimized independently but rather a link deeply embedded in the entire production and operation system of the enterprise. Upstream decisions (such as process planning and product design), downstream decisions (inventory management and material supply), and decisions at the same level (such as workshop logistics and worker scheduling) are all closely coupled with and mutually influence the balance of the production line [16,17,18]. Therefore, isolated research on production line balancing can no longer meet the needs of system optimization.

Based on this evolutionary trend from “isolated problems” to “system integration,” this paper aims to systematically review how issues related to production line balancing have gradually expanded their research boundaries from a defined core problem by continuously absorbing new constraints and objectives and how they have deeply integrated with other production management decision-making areas. Unlike existing reviews that mostly focus on a certain type of problem or a specific solution method, we construct the C|H|V|E model to depict the research expansion path of the production line balancing problem in a systematic way.

(1) Advantage 1: Panoramic and Structured Framework: The C|H|V|E model organizes the literature in four evolutionary dimensions—C (core issue complexity), H (horizontal integration with shop-floor decision-making), V (vertical integration with enterprise-level decision-making), and E (value dimension extension covering efficiency, sustainability, and resilience). The framework provides a structured tool for understanding the intrinsic connections between the different branches.

(2) Advantage 2: Evolutionary Logic: Unlike simple static classification, this model highlights how LBP gradually evolves from a well-defined optimization problem to a system engineering challenge involving multiple levels of production management.

(3) Advantage 3: Future-oriented: By revealing the extended trajectories and integration paths of problematic research, we identify the intersection of LBP with emerging technologies (e.g., artificial intelligence, digital twins, human-machine collaboration, and full lifecycle integration optimization) and future research opportunities.

Therefore, the novelty of this study lies not only in the comprehensive coverage of the literature, but also in the proposal of a new conceptual model (C|H|V|E), which can help the academic community to understand the development of the field from a panoramic perspective and provide some guidance for future research.

The structure of this paper is as follows: Section 2 provides an overview of the research methods used for research selection, extraction, and adoption and conducts a statistical analysis of the literature to identify current research hotspots and trends in this field. Section 3 analyzes the literature from the C perspective, revisiting the deepening of core issues, namely the complex evolution of LBP itself in terms of layout, models, and task characteristics. Section 4 and Section 5 explore its integrated development from the H and V perspectives, respectively. The former focuses on collaboration with other workshop decisions (such as scheduling and worker allocation), while the latter examines integration with other aspects of enterprise operations (such as process planning and supply chain management). Section 6 will provide a commentary from the E perspective, analyzing the expansion of its value dimensions, specifically how optimization objectives have evolved from a single efficiency-oriented approach to a multi-dimensional value system incorporating sustainability and resilience. Finally, Part 7 summarizes the full paper and looks forward to future research directions and challenges from a system integration perspective.

## 2. Literature Review

### 2.1. Literature Selection

This review explores the expansion of the boundaries of production line balancing issues from an “integrated” perspective, aiming to reveal the intrinsic connections between different branches and speculate on the development trends in cross-disciplinary fields. Therefore, the literature search focuses on “production line balancing” while also considering the breadth and systematic nature of the search scope.

**Figure 1:**
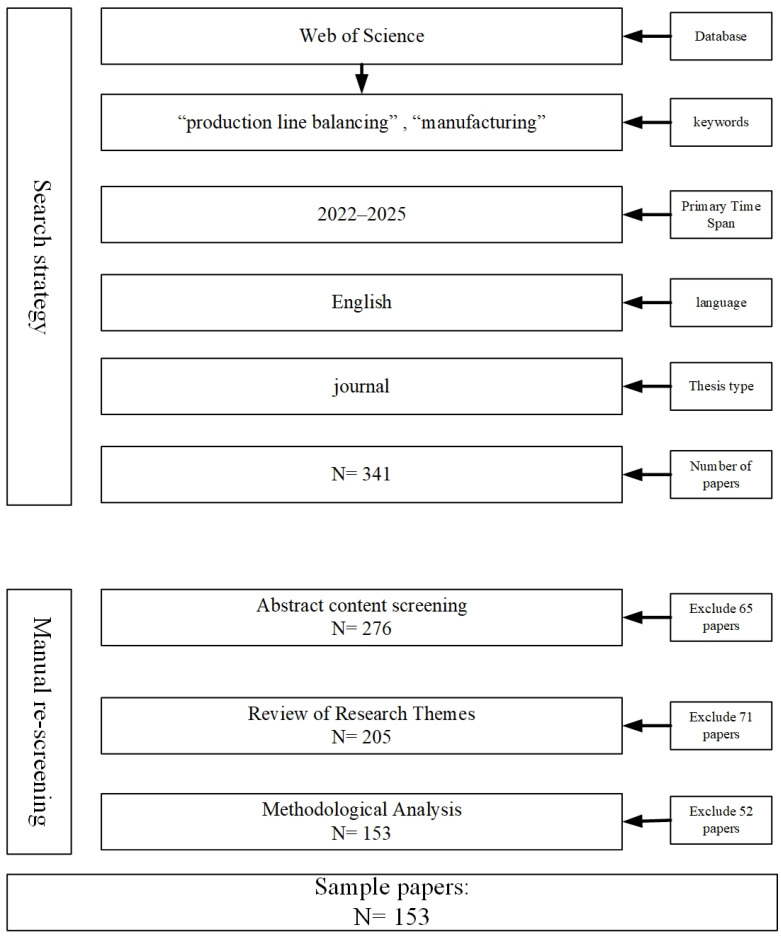
Literature selection process.

**Figure 2:**
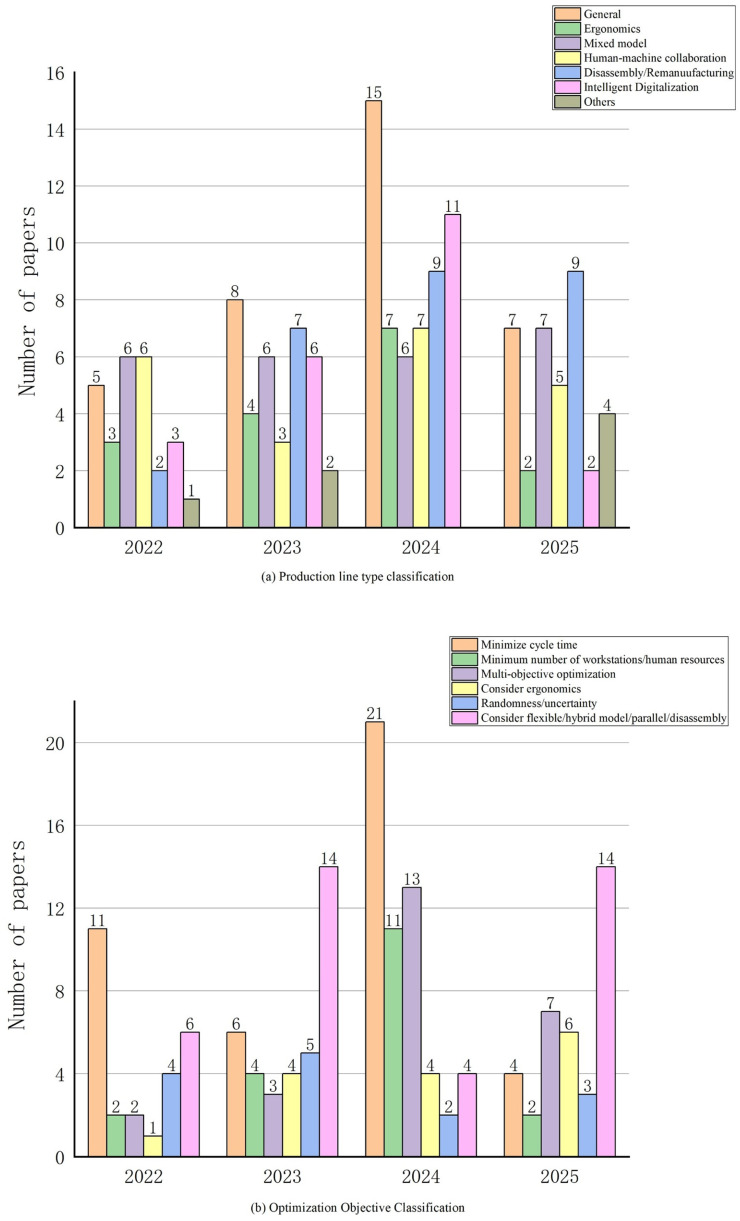
Distribution of selected literature by year and research direction.

This study is based on the Web of Science (WoS) database for literature search and analysis. The main reason for choosing WoS is that it has high academic authority and coverage in the fields of production management and operation research optimization, and the literature included has been strictly peer-reviewed, which can guarantee the quality of data and the reliability of research results. Meanwhile, WoS provides standardized citation information and complete search tools, which facilitates systematic and repeatable bibliometric analysis. Compared with other databases, WoS has advantages in literature standardization and data consistency and is therefore widely used in review and knowledge graph research. The search strategy consists of two “AND” operators, focusing on two keywords “line balancing” and “manufacturing”. The search was limited to English-language documents, journal articles, and the time span of 2022–2025. After manually reviewing the abstracts, organizing the research themes and methodologies, and other steps, a total of 153 relevant articles were selected as the analysis sample. Figure 1 illustrates the literature screening process; Figure 2 presents the distribution of the literature across the two research directions based on the statistical analysis of the number of documents from different years:

(I) By production line type: general assembly lines, ergonomically designed assembly lines, mixed-model production lines, human-machine collaborative assembly lines, disassembly/remanufacturing production lines, intelligent digital production lines, and other specialized production lines;

(II) By optimization objectives: minimum production cycle, minimum number of workstations/human resources, multi-objective optimization (time, cost, energy consumption, carbon emissions), ergonomics considerations, randomness/uncertainty considerations, and flexibility/hybrid model/parallel/disassembly production lines.

### 2.2. Literature Analysis

**Figure 3:**
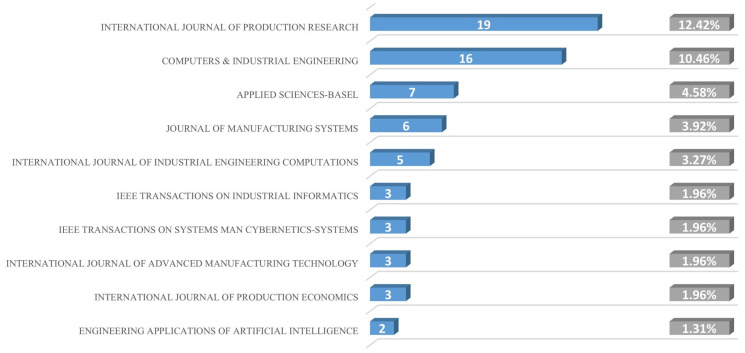
Ranking and percentage of journal publications.

This paper uses a combination of literature statistics and content analysis to conduct a preliminary analysis of the selected literature, laying the foundation for the core issue research, horizontal integration and vertical integration analysis, and value dimension expansion in the latter part of this paper. Figure 3 shows the names and numbers of articles from the top 10 journals in this field, as well as their percentage of the total number of articles published.

#### 2.2.1. Keyword Co-Occurrence Analysis

**Figure 4:**
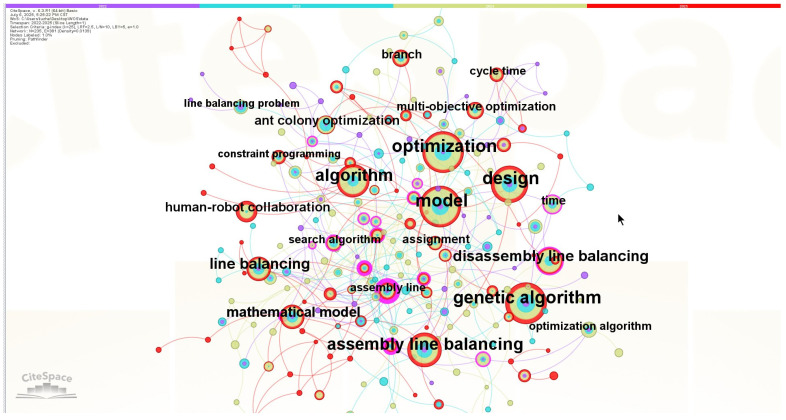
Keyword co-occurrence map.

This review employs CiteSpace 6.3.R1 (64-bit) software to conduct co-occurrence analysis of keywords within selected literature, aiming to reveal current research hotspots in this field through the frequency and temporal patterns of keyword appearances. In the knowledge map, nodes stand for keywords, and the size of the nodes shows how often they appear. The thickness of the lines between nodes shows how strong the connection is, and the color shows when the publication was made. Based on the analysis results of literature from the Web of Science database from 2022 to 2025 (Figure 4), a total of 235 nodes and 381 connections were obtained. Among them, “model” and “optimization” had the highest occurrence frequencies, followed by “genetic algorithm,” “design,” “assembly line balancing,” and “algorithm.” Other visible keywords also had occurrence frequencies exceeding 10 times.

Table 1 lists the high-frequency keywords in the field of production line balancing. The results of the co-occurrence analysis presented in Table 1 and Figure 4 can be summarized as follows:

(1) Research hotspot. Research in this area is still centered on modeling and optimization, with “model” (108 times) and “optimization” (107 times) appearing most frequently, followed closely by “genetic algorithm” (100 times), “design” (91 times), “assembly line balancing” (74 times), and “algorithm” (74 times). This indicates that the focus of assembly line balancing research continues to be on how to improve productivity through modeling and optimization methods and to cope with complex constrained environments with the help of genetic algorithms and heuristics. In terms of centrality, “assembly line” (0.43), “job rotation” (0.31), “artificial bee colony algorithm” (0.30), and other keywords have a strong bridging role in the network and constitute an important hub for knowledge exchange. These nodes indicate that assembly line balancing and task allocation are still the core issues in the field; job rotation and human factors are gradually becoming a research hotspot, which indicates that the research is shifting from pure efficiency orientation to the direction of taking into account human resources and sustainability; and the application of group intelligence algorithms such as the artificial bee colony algorithm and the particle swarm optimization highlights the position of intelligent optimization methods in solving complex problems.

(2) Research trends. Combined with the analysis of research development from 2022 to 2025, it is easy to see that early research emphasized mathematical modeling and traditional optimization algorithms. However, with the gradual complexity of the production environment, intelligent optimization algorithms (genetic algorithms, artificial bee colony, particle swarm, etc.) have become the main research direction. In the last two years, human factors and human-robot collaboration (human-robot collaboration, job rotation, worker assignment) have gradually become emerging keywords in research, suggesting to a certain extent that the research in this field is expanding to smart manufacturing and social sustainability.

(3) Research redundancy and gaps. Although there have been a large number of studies on production line balance optimization, there are still deficiencies in some aspects. There is a certain saturation of research in optimizing the production cycle of production lines, and methods such as genetic algorithms, ant colonies, particle swarms, etc. have been widely studied, showing a trend of algorithmic redundancy. However, research in certain emerging directions is still insufficient, such as human-machine collaboration, worker comfort, flexible manufacturing, and other topics involving smart factories that are still in their infancy. Meanwhile, the interdisciplinary integration in this field is limited, and integration with machine learning, digital twin, and real-time data-driven still needs to be strengthened so that data-driven dynamic production line balancing models can be explored in the future.

#### 2.2.2. Keyword Cluster Analysis

**Figure 5:**
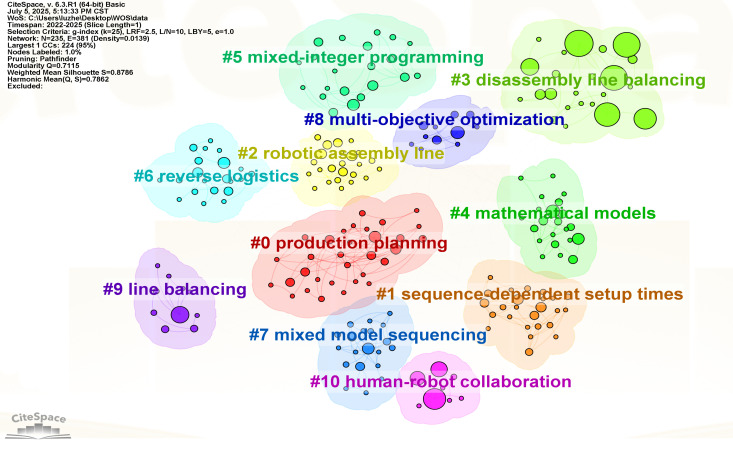
Keyword Cluster Analysis Chart.

The cluster analysis shows that the Q-value is 0.7115, which is more than 0.3, and the S-value is 0.8786, which is more than 0.7. This indicates that the network structure exhibits significant modularity, confirming the reliability of the analysis results. A total of 11 clusters were identified (Figure 5), and there was a certain degree of correlation between them. The main keywords are listed in Table 2.

**Figure 6:**
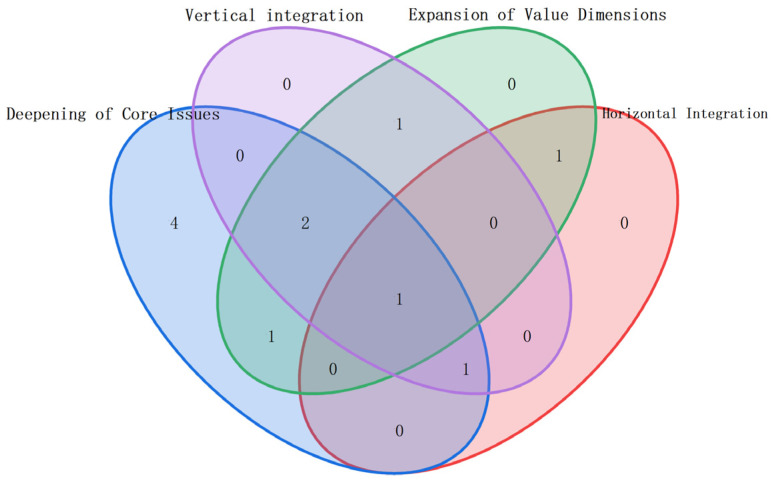
C|H|V|E-Clustering Results’ Classification.

The cluster analysis shows overlaps among clusters. Using the C|H|V|E framework (Table 3), the results are categorized into four dimensions: C, H, V, and E, with their relationships illustrated in a Venn diagram (Figure 6). The following sections analyze the literature and future directions from these four perspectives.

## 3. C: Increased Internal Complexity

The classic SALBP has laid the foundation for research in this field. However, to better align with real-world production environments, research must overcome the idealized assumptions of SALBP and incorporate additional practical constraints, thereby significantly increasing the internal complexity of the problem. This refinement is primarily manifested in three aspects: production line layout, objective diversification, and task uncertainty [19,20,21], as shown in Table 4, which will be further elaborated upon in subsequent sections.

### 3.1. Diversified Production Line Layout

Traditional production line research often assumes a single linear layout, with workpieces flowing through each workstation in a fixed order. To improve space utilization, enhance worker collaboration, and shorten material handling distances, various nonlinear layouts have emerged, each of which poses new challenges for production line balancing. This article focuses on U-shaped line balancing problems (ULBP), two-sided assembly line balancing problems (TSALBP), and parallel line balancing problems (PLBP). The U-shaped layout places the production line entrance and exit adjacent to each other, enabling workers to operate multiple workstations simultaneously [45]. The complexity of ULBP arises from the fact that task allocation is no longer confined to a singular direction; a single workstation may encompass tasks from various production stages, thereby significantly broadening the solution space [23] and rendering conventional unidirectional heuristic rules ineffective. To this end, Jiao et al. [46] constructed a multi-objective model with the minimum smoothing index and number of workstations as the objectives and proposed an improved ant colony algorithm that integrates heuristic factors and pheromone information. In response to the dynamic disturbances of actual U-shaped disassembly lines, Wang et al. [47] introduced deep Q-networks (DQN) to achieve dynamic equilibrium. In TSALBP, paired workstations must meet the requirements for process sequence, operation direction, and load balancing at the same time [48]. This system adds more spatial and synchronization constraints, making it much more complicated than single-line systems. Large product assembly often requires simultaneous operations on both sides of the production line. As an example, in the case of an automotive mixed-flow two-sided assembly line, Liao et al. [49] proposed a model without observation point restrictions to describe the vehicle model’s sequence, number of workstations, and task allocation and designed an improved genetic algorithm that combines combination and evaluation mechanisms to improve balance and sorting efficiency. In practical applications, preventive maintenance can lead to production line downtime and wasted capacity; so, it is necessary to preset multiple interchangeable task allocation schemes to adapt to different scenarios. Zhao et al. [50] to solve this kind of problem, it constructed a multi-objective mixed-integer planning model and also proposed a knowledge-assisted variable neighborhood search (KVNS) method, which minimizes the cycle time while reducing the amount of task adjustment. When single-line production capacity is insufficient, companies often deploy multiple parallel production lines (PLBP), which not only require optimizing internal balance across lines but also cross-line task allocation to maximize output or minimize costs, involving higher-level load balancing decisions, and the problem scale grows exponentially. Zhang et al. [51] proposed a parallel two-sided disassembly line balancing problem with fixed shared workstations, established a multi-objective mixed integer programming model, and used a multi-objective firefly algorithm to solve it. Mao et al. [52] explored the application of human-machine collaboration in parallel assembly lines and its potential to improve efficiency and resource utilization. To optimize the profit and disassembly time of multi-product parallel disassembly lines, Guo et al. [53] constructed mathematical models and multiple correlation matrices and designed a multi-objective discrete chemical reaction optimization algorithm, which was verified for its feasibility and superiority on ballpoint pen, flashlight, washing machine, and radio assembly lines.

### 3.2. Optimization Objective Changes

SALBP usually assumes that a production line only makes one type of product. However, current market demands for diverse customization require production lines to simultaneously produce multiple products [54], giving rise to multi-objective and mixed-objective balancing problems [55]. The Multi-Objective Assembly Line Balancing Problem (MOALBP) addresses changeover issues that arise when producing multiple products in batches. The goal is to simultaneously optimize multiple conflicting performance metrics to meet the combined requirements of efficiency, flexibility, and cost [56]. In today’s era of heightened carbon footprint awareness, Tao et al. [57] propose a multi-objective optimization model that accounts for worker skill levels to address the challenge of balancing productivity, emissions, and profits. The Mixed-Model Assembly Line Balancing Problem (MMALBP) refers to the production of multiple products on the same assembly line in arbitrary sequences [43]. Research on this problem centers on how to efficiently produce multiple products while satisfying process logic and capacity constraints [58].

### 3.3. Changes in Production Line Processes

SALBP usually assumes that the task time duration is fixed, but this is idealized. Because in the actual production process it is affected by worker proficiency, fatigue, equipment failure, and material fluctuations. To more accurately represent this uncertainty [59], research introduces random variables governed by specific probability distributions, resulting in the Stochastic Assembly Line Balancing Problem [60]. The primary objective is to minimize the number of workstations or cycle time at a designated confidence level. For example, the study of the Robotic Stochastic Assembly Line Balancing Problem (RSALBP) is usually to optimize the production cycle time of a product under the assumption that the task time follows a normal distribution with a defined number of stations and robots [61]. When there is a lack of sufficient time data, some researchers have used interval type II fuzzy set theory to solve the problem [41]. In some assembly processes, process time is affected by preceding processes. Preparatory activities such as tool replacement, fixture adjustment, or position adjustment can change the operation time and increase the complexity of task allocation. In mixed-flow robot two-sided assembly line balancing, considering sequence-related preparation time is crucial to minimizing the production cycle [62]. Under two-sided resource constraints, process priority constraints must also be included [63]. Research has also expanded to complex tasks such as multi-task allocation, multi-worker collaboration, and material distribution, proposing an integer linear programming model and a heuristic algorithm based on adaptive large neighborhood search (ALNS) to minimize the total system costs of warehousing, transportation, assembly, and investment [44].

## 4. H: Collaborative Optimization with the Workshop Level

After fully understanding the internal complexity of production line balancing, the research perspective needs to be expanded to the workshop level. Production line balancing is not an isolated static plan but is closely coupled with other dynamic, real-time decisions at the same level. Isolated optimization can fall into the dilemma of “local optimum, global sub-optimum.” Therefore, integrating it with related decisions horizontally to achieve collaborative optimization is the key to improving the overall operational efficiency of the workshop (shown in Table 5 and below).

### 4.1. Integration with Product Sequencing

According to the definitions mentioned in Section 2, the combination of line balancing and product production sequencing is a typical lateral integration problem. This type of problem is more obvious in mixed-flow production lines. This is because in a mixed-flow production line, different types of products can be produced at the same station. Whenever the production of a particular product reaches an optimal equilibrium, the line equilibrium will not be maintained after the line change, and overloading of the stations may occur [72]. Therefore, some researchers have raised the issue of hybrid model balancing and ranking [73]. They simultaneously address uncertain task durations by employing an improved genetic algorithm and establishing a distributed robust optimization model to optimize task sequencing and reduce production cycles [74]. Typically, production managers must address issues such as task allocation, product sequencing, job completion times, and delivery deadlines when formulating production plans [75]. They generally prefer to simultaneously optimize task allocation and product sequencing within a unified framework to minimize the number of workstations and overall costs. This method often relies on real-time manufacturing data to build a dynamic rebalancing framework and combines improved intelligent algorithms (such as the two-stage adaptive alternating genetic fireworks algorithm) to dynamically adjust task sequences, thereby enhancing the robustness of production lines and their adaptability to dynamic product flows [76].

### 4.2. Integration with Worker Assignment

Traditional LBP rarely concern workers. Nonetheless, in actuality, there are objective disparities among workers regarding abilities, competence, and efficiency, referred to as worker heterogeneity [77] Production line balancing issues combined with worker allocation seek to attain synchronization between personnel and production activities [78]. Li and Wang [79] endeavored to develop an integer programming model for workstations in human-robot collaborative assembly lines, utilizing an enhanced particle swarm optimization technique to refine the distribution of tasks between humans and robots. Learning and forgetting effects also affect operating hours, and in response to the worker allocation problem, Perez-Wheelock et al. [80] considered the effects of learning and forgetting curves to develop a rebalancing model. Alhomaidhi [81], in order to solve the mixed-flow production line equilibrium problem, blended learning effects and worker prerequisites to optimize the task allocation in the assembly line, which improved the resource utilization of the production line. With the rise of the human-centered concept, the manufacturing industry has begun to pay attention to the welfare of workers, and more research has been conducted in this regard. For example, long hours of repetitive labor and incorrect working postures can cause certain injuries to workers and also affect productivity. Abdous et al. [82] considered ergonomics related content in the study of production line balancing. Kulac and Kiraz [83] on the other hand, in the field of hybrid assembly line research, took into account the production cycle and human factor. Tiacci [84] proposes a combination of discrete time and genetic algorithms to allocate reasonable rest periods to workers engaged in ergonomically risky tasks on the assembly line from the perspective of guaranteeing the profitability of the company. Noda et al. [85] developed an optimal allocation model for intelligent production lines, balancing quality and delivery under limited cycle and multi-stage conditions. In order to solve the problem of labor stratification and cost constraints in production line balancing, Kang et al. [86] proposed a multi-objective balancing and hierarchical worker assignment model. Zeng et al. [87], on the other hand, optimized the task time allocation of the human-machine collaborative production line by designing a heuristic search algorithm, which efficiently balanced the production efficiency and workers’ fatigue. In order to safeguard workers’ fatigue recovery and reduce their risk of skeletal diseases, Abdous et al. [88] balanced workers’ fatigue recovery and task allocation through an iterative bifurcated search method.

### 4.3. Integration with Material Handling

The balance and efficiency of the production line on the shop floor is largely dependent on a stable and timely material supply [71]. The layout of the production line stations is also limited to some extent by the material delivery strategy. For a customized printed circuit board (PCB) assembly line, Mumtaz et al. [89] proposed a genetic-artificial bee colony algorithm to optimize task sequences and Automated Guided Vehicle (AGV) material handling paths. To balance the material supply of the assembly line, Arik and Yufka [90] constructed a mixed integer planning model to improve productivity. Integrated research on material handling and line balancing aims to coordinate optimization of feeding schemes and task allocation. Assigning tasks with similar requirements to adjacent workstations reduces material handling paths. To optimize the material handling problem in a flexible job shop, Hou and Zhang [91] proposed an improved multi-objective whale optimization algorithm to optimize AGV loads and multiple AGVs for cooperative handling. With the application of various advanced technologies in production systems, the LBP is gradually integrated horizontally with decisions made at the shop floor level, such as product sequencing, worker assignment, and material handling [92], so that it is no longer a closed problem but an open and dynamically coordinated process.

## 5. V: Collaborative Optimization at the Enterprise Operational Level

Compared with horizontal integration that focuses on coordination with the shop floor, vertical integration emphasizes the pivotal role of production line balancing in the enterprise value chain, as described in Table 6 and below. Production line balancing not only carries the results of upstream product design and process planning but also directly affects downstream production planning and supply chain operations. Therefore, breaking down the vertical barriers between production line balancing and operational decision-making and achieving vertical coordination optimization is of enormous strategic significance for enhancing the overall competitiveness of the enterprise.

### 5.1. Upstream Integration: Design and Process

Integrating balancing issues with upstream decisions can create solutions that are more conducive to efficient balancing from the outset, with product design and process planning having a particularly significant impact. Typically, research on assembly line balancing issues can only be conducted on existing production lines. Whereas Alfaro-Pozo and Bautista-Valhondo [93] found that it is possible to base the need for line balancing on considerations at the design stage, advocating the consideration of design for assembly (DFA) issues. Additionally, methods such as modular design to reduce component count, tooling utilization, and the design of foolproof interfaces can shorten product assembly time, optimize subsequent balancing issues, and positively contribute to lowering manufacturing costs [103]. Process planning strives to transform product designs into operational manufacturing processes. Traditional line balancing studies typically assume that the process path is unique and fixed [104], but from an integrated perspective, different process paths generate different task sets, directly affecting balancing efficiency. In studies concerning disassembly line issues, it is widely recognized that predictable process planning facilitates the modeling of workshop equipment layout and LBP [94]. Therefore, integrating process planning with production line balancing research not only resolves line balancing challenges but also identifies the optimal solution among multiple process route options to maximize production line balancing efficiency. Although this approach increases decision-making complexity, it also provides a broader scope for finding optimal solutions to global problems.

### 5.2. Downstream Integration: Supply Chain and Warehousing

The takt time and flexibility of production lines also provide crucial information inputs for downstream operational decisions, while exerting a certain influence on supply chain responsiveness [105]. A stable and efficient production line is a prerequisite for lean production and just-in-time (JIT) supply. In the field of supply chain optimization research on frozen products, Kittichotsatsawat et al. [100] used lean manufacturing methods such as value stream mapping and ECRS (Eliminate, Combine, Rearrange, Simplify) to improve the production process and increase the efficiency of the frozen products production line. Also worthy of our attention is the balance of the production line, which affects the level of work-in-process (WIP) inventory. Specifically, when a production system is balanced, the flow of materials through the production line is smooth, and the level of WIP inventory is low; when there are bottlenecks in the production line, there is often waiting and a buildup of WIP, which corresponds to an increase in inventory costs. Upstream signals of material demand are directly impacted by the stability and predictability of production lines. Through the bullwhip effect, unstable signals typically increase demand uncertainty, compelling supply chain segments to boost safety stock and reducing overall profitability. Kampa and Paprocka [101] proposed a method to shorten the production line and introduce a U-shaped line with a cellular layout. At the same time, he proposed to set up work-in-progress inventory in critical areas to enhance the stability of the production line. Through the above analysis, we can conclude that the study of production line balancing optimization problem not only improves the productivity of the shop floor but also makes the supply chain more stable and plays an important role in the reduction in the total cost of the production system. The vertical integration of production line balancing with upstream and downstream decision-making has become a strategic area of research, significantly impacting product design and company operations [102], while this systematic vertical thinking also broadens the scope of the research.

## 6. E: From Efficiency to Sustainability and Resilience

With the rise of global economic development and sustainable development concepts, the research objectives for production line balancing have quietly shifted. The focus has moved from traditional goals like minimizing production cycles, maximizing efficiency, reducing production costs, and increasing output to a multi-objective optimization approach that prioritizes environmental and sustainable development. Production lines that sacrifice environmental integrity or employee well-being to boost efficiency, or those unable to withstand market fluctuations, lack resilience and sustainability. Such lines are not favored. Relevant literature on the E dimension is summarized in Table 7 and discussed below. Therefore, the value orientation of production line balancing is expanding from traditional efficiency goals to two new dimensions: sustainability and resilience.

### 6.1. Sustainability

Sustainability emphasizes balancing economic benefits with environmental and social responsibility, a concept that has driven the expansion of production line balancing research into energy and human factors [111,115]. Previously, energy consumption was usually fixed in production line balance optimization. Nowadays, more and more attention has been paid to the fact that different scheduling schemes have different impacts on energy use in the shop floor [116]; so, the research on balance optimization of energy consumption has gradually become a hot topic. It is worth noting that researchers usually study energy consumption, emission, and efficiency together as multiple optimization objectives. For example, to reduce the energy consumption of idle equipment in the workshop and improve the production efficiency, Sun et al. [117] designed a bi-objective optimization model for a human-robot mixed-flow assembly line, which reduces the production energy consumption by using an improved cuckoo search algorithm and shutdown strategy, while Elmolouk et al. [118] optimized the production cycle time and the energy consumption of robots by using an integer programming model, and Chi et al. [119] proposed a hybrid integer programming and simulated annealing algorithm in cross-station task scenarios, prioritizing workstation minimization while also minimizing energy consumption, thereby balancing efficiency and green objectives. In the context of a circular economy, the dismantling line balancing problem (DLBP) has become a hot topic of research, with the goal of maximizing efficiency and profitability in product recycling and remanufacturing [120]. Compared to assembly lines, DLBP faces more uncertainties, such as the condition of parts and rusting [121], while also needing to balance economic and environmental benefits. Recently, human factors have also received much attention from experts in the field of production line balance. Guo et al. [122] and Wei et al. [123] added the standing and sitting postures of the workers during operation to the optimization model, which was solved with the multi-objectives of profit maximization and labor intensity minimization using the Pareto-based harmonic search algorithm. Qi et al. [124] paid more attention to the research in the remanufacturing field (e.g., heterogeneous plants and dismantling lines) by constructing a mixed-integer programming model and applying a strategic gradient algorithm to it. The problem is solved. In a study on human-robot collaborative disassembly line equilibrium, Cui et al. [125] designed a whale optimization algorithm based on evolutionary learning, which has a better performance in dealing with large-scale disassembly instances, and Zacharia, Xidias, and Nearchou [126] designed a meta-heuristic algorithm to explore the solution for the variation and uncertainty of workers’ operation time in collaborative robot production lines. a metaheuristic algorithm to explore the solution. It is worth noting that the long-term development and health of employees are also gaining attention from researchers. Kheirabadi et al. [127] point out that line balancing can be used to optimize job design, while Nourmohammadi, Fathi, and Ng [112] emphasize the promotional effects of human-machine collaboration and job rotation on skill development and job satisfaction, thereby fostering a learning workforce and enhancing overall efficiency.

### 6.2. Resilience

The ability of a production line to adapt and recover in the face of internal perturbations and external shocks is usually referred to as the resilience of the production line [128,129]. Efficient but fragile production lines are of limited value in dynamic environments, and thus resilience is becoming an important goal in production line balancing decisions. Research focuses on maintaining system operation under localized failures through redundant designs and flexible paths. Resilience-oriented balancing schemes emphasize the maintenance of controllable performance under failure conditions to provide robust operation of the production system. Resilient production lines rely on highly flexible staffing. Employee rotation training helps them to quickly adapt to multiple letting tasks, which can reduce the risk of production disruptions to some extent. Albus et al. [130] argue that research on production lines should focus on their resilience and reconfigurability to external shocks, and that production schemes should be designed to allow for rapid switching and capacity adjustments to respond to changing market demands [131]. Related research is devoted to designing schemes that combine current efficiency with future rebalancing capabilities to achieve line adjustments at the lowest cost and time [113,132]. In distributed manufacturing and reconfigurable production environments, different shops are required to produce multiple products, but research on optimizing line balancing and reconfiguration integration in distributed factories is still limited. For the reconfigurable production line balancing optimization problem in distributed manufacturing systems, Yang et al. [114] proposed a dynamic rescheduling and iterative greedy algorithm based on meta-heuristics and also optimized the scheduling and reconfiguration of dynamically distributed reconfigurable flow shops. Meanwhile, the objective of production line balance optimization gradually shifts from static to total cost minimization over the whole life cycle. To guarantee the stable operation of production and no interruption of production in case of preventive maintenance, the researchers proposed multiple sets of task allocation plans. When a total of one plan needs to be suspended due to maintenance, it can be switched to the other plans to guarantee the production continuity. Tang et al. [133] proposed in this regard that the coupling relationship between different plans should be considered when doing the production line balancing optimization. To this end, he proposed a multi-objective, multi-factor evolutionary algorithm that is able to treat the assembly line balancing problem under each scheme as a multi-tasking optimization problem in order to minimize the production cycle time and the line adjustment time to guarantee the production continuity. In order to solve the problem of insufficient toughness of the production line, Wang et al. [134] proposed a rolling dispatch and production line balancing mechanism, which combined with a two-stage genetic algorithm to generate a production line balancing scheme with toughness, and designed an efficient line-changing mechanism to reduce the energy consumption of the machine operation and standby and realize green production.

## 7. Discussion and Future Research Directions

### 7.1. Discussion

Since Frederick Winslow Taylor established the concept of “standardized work” by measuring standard times for each process step in the creation of the Ford Model T assembly line, the issue of production line balancing has undergone over a century of evolution. It has evolved from a combinatorial optimization problem with a single objective to a complex system engineering challenge that integrates operations research, computer science, and human factors engineering [135,136]. A brief review of its developmental history reveals several core evolutionary pathways:

(1) Increasing model complexity. Optimization models have progressively incorporated various constraints encountered in actual production processes—such as production line layout and worker heterogeneity—since the initial SALBP framework. This integration has brought researchers’ optimization models closer to real-world production scenarios [137].

(2) Decision-making has shifted from isolation to integration. Decision problems in production line balancing optimization have expanded beyond internal production line issues to include horizontal coordination at the workshop level (e.g., material handling) and vertical integration into enterprise operational management (e.g., product design, supply chain management). This arrangement also avoids, to some extent, the limitations of decision-making [138].

(3) From singular to multifaceted: Production lines’ value pursuit has expanded beyond pure economic efficiency to encompass sustainability—including environmental impact and employee well-being—alongside resilience against internal and external disturbances. This has established a more comprehensive and forward-looking optimization objective system [139].

In summary, the contribution of this paper is to provide an integrated perspective on the evolutionary path of production line balancing beyond the limitations of traditional reviews that focus on a single issue or methodology. By constructing a four-dimensional analytical framework of C|H|V|E, this paper not only systematically presents the expansion of the research boundaries and the intrinsic connections among branches but also provides a structured tool for academics to understand the overall evolutionary logic of the field and to identify future research opportunities. In an academic sense, this study provides a panoramic cognitive framework for the study of production line equilibrium problems, which helps to promote its transformation from an isolated optimization problem to a multidimensional system engineering challenge.

However, there are some limitations in this study: Firstly, the literature data mainly come from the Web of Science database, which is representative and authoritative, but it is difficult to cover all the relevant research results; secondly, the C|H|V|E framework proposed in this paper is mainly used for systematization of the theoretical level, and its effectiveness and applicability in practical application still need to be further verified through subsequent empirical research and case studies. In addition, this paper does not discuss the implications for policy and marketing, nor the economic feasibility. Future research can further analyze the potential value of the balanced approach of intelligent and sustainable production lines in policymaking and corporate strategy and evaluate the trade-off between efficiency improvement and implementation cost in combination with actual cases to better promote the combination of theory and practice in this field.

### 7.2. Future Research Directions

In the context of Industry 4.0 and smart manufacturing technologies, the LBP is not only not obsolete but also given a new connotation and a broader research space. Future research is no longer just an update and extension of the existing problem model but is more likely to be a breakthrough innovation driven by new technological paradigms [140,141,142]. The following key directional areas of research can be considered in the future.

(1) Intelligent and data-driven technology integration. The deep integration of Artificial Intelligence (AI), the Internet of Things (IoT), and Digital Twin (DT) will become an important development direction for production line balance. On the one hand, with the help of IoT and wearable devices, massive data such as workstation load, equipment status, and workers’ physiological indexes can be collected in real time, and combined with machine learning and reinforcement learning algorithms, we can realize the transformation from “static planning” to “dynamic predictive balancing” [143,144]. For example, the system can predict potential equipment failures and adjust task assignments in advance [145] or dynamically adjust workloads based on real-time worker fatigue. On the other hand, digital twins provide a closed-loop “physical-virtual-decision-control” optimization mechanism for production lines [146,147]. Researchers can simulate and stress test different equilibrium scenarios in a virtual environment to evaluate their resilience and efficiency and then send the optimization results to the physical production line in real time [148], resulting in an adaptive and self-learning production system.

(2) Flexible balance of human-machine collaboration and ergonomic orientation. With the rapid development of industrial robots and collaborative robots, the future production line will show the trend of human-robot coexistence and collaborative work [149]. This trend will lead to a new equilibrium problem, i.e., how to realize flexible task allocation between workers and robots. The research needs to consider not only the traditional working time but also the safety, smoothness, and ergonomics of human-robot interaction [150,151]. In the future, it will be necessary to explore the dynamic matching mechanism of task-resource (human/machine) [152] to maximize the efficiency of human-computer collaboration [153] and to build safe, comfortable, and flexible production cells.

(3) Sustainability and resilience-driven system optimization. In the context of green manufacturing and circular economy, the study of production line balancing needs to break through the limitations of “shop floor optimization” and move towards “maximization of whole life cycle value.” This requires the systematic integration of Design for Assembly (DFA), Design for Manufacturing (DFM), and Design for Disassembly/Recycling (DFD/Design for Recycling, DFR). At the beginning of product design, a balanced model should be used to assess the combined impact of different options on subsequent assembly, disassembly, assembly, and remanufacturing. In addition, future production systems must be more resilient to uncertainties such as emergency order insertion, worker absenteeism, equipment failures, and supply chain disruptions to improve the robustness and long-term resilience of the production system.

To summarize, the essence of the production line balancing problem is to make the operating time of each station as balanced as possible through the reasonable distribution of process tasks so as to reduce the waste of resources and loss of efficiency. However, future research should not stop at optimizing a single physical production line but should be geared toward building a more intelligent, humanized, sustainable, and resilient manufacturing system. In this evolutionary process, line balancing, as the cornerstone of industrial production, will continue to play an irreplaceable core role, and with the continuous integration of technology and cross-domain fusion, it will continue to revitalize new vitality.

## Figures and Tables

**Table 1:** High-frequency keyword ranking in the field of line balancing.

Word Frequency Sorting			Centrality Sorting		
Number	Count	Keywords	Number	Centrality	Keywords
1	108	model	1	0.43	assembly line
2	107	optimization	2	0.31	job rotation
3	100	genetic algorithm	3	0.3	artificial bee colony algorithm
4	91	design	4	0.2	artificial bee colony
5	74	assembly line balancing	5	0.17	network design
6	74	algorithm	6	0.16	mixed-integer programming
7	45	disassembly line balancing	7	0.14	parallel workstations
8	41	line balancing	8	0.13	worker assignment
9	35	mathematical model	9	0.13	particle swarm optimization
10	27	human-robot collaboration	10	0.12	search algorithm

**Table 2:** Cluster Group Keyword Table.

Cluster ID	Cluster Name	Size	Silhouette	Label (LLR)
#0	production planning	32	0.859	production planning, industry 4.0, mixed-model assembly line balancing, ergonomics, disassembly line balancing
#1	sequence-dependent setup times	25	0.907	sequence-dependent setup times, two-sided assembly line balancing, artificial bee colony algorithm, local search, simple assembly line balancing
#2	robotic assembly line	24	0.874	robotic assembly line, heuristic algorithms, data validation problem, domain generalization, decision support systems
#3	disassembly line balancing	23	0.864	disassembly line balancing, disassembly planning, green manufacturing, collaborative robots, sustainable manufacturing
#4	mathematical models	22	0.896	mathematical models, workstations, search problems, layout, costs
#5	mixed-integer programming	21	0.811	disassembly line balancing, mixed-integer programming, assembly line balancing, recursive approach, human-robot interaction
#6	reverse logistics	21	0.841	reverse logistics, chance-constrained programming, reconfiguration, decomposition heuristic, joint assembly line balancing and feeding problem
#7	mixed model sequencing	20	0.893	mixed model sequencing, task sharing, reconfigurable manufacturing systems, classification scheme, dynamic line balancing
#8	multi-objective optimization	11	0.896	multi-objective optimization, robotic assembly line balancing, industry 4.0, stochastic assembly line balancing
#9	line balancing	10	0.872	line balancing, lean manufacturing, lean manufacturing, multi-objective optimization
#10	human-robot collaboration	8	1	human-robot collaboration, resource sharing, u-shaped assembly line, hybrid disassembly line balancing, mathematical model

**Table 3:** C|H|V|E Analysis Criteria.

Criteria	Contents	Cluster ID
C Deepening of core issues	Section 3.1 Layout expansion: from linear to U-shaped, two-sided, and parallel lines Section 3.2 Objective expansion: From single objective to mixed/multi-objective Section 3.3 Process uncertainty: from deterministic to random/fuzzy time; from simple to sequence-dependent	#0, #1, #4, #5, #6, #7, #8, #9, #10
H horizontal integration	Section 4.1 Integration with Product Sequencing: Sorting Issues in Mixed-Flow Production Lines Section 4.2 Integration with Worker Assignment: Skills, fatigue, learning curves, etc. Section 4.3 Integration with material handling: feeding methods and costs	#2, #6, #10
V vertical integration	Section 5.1 Upstream integration: product design, process planning Section 5.2 Downstream integration: integration with warehousing and supply chain (especially dismantling line balancing in reverse supply chains)	#0, #3, #5, #6, #10
E Expansion of value dimensions	Section 6.1 Sustainability: energy consumption, carbon emissions, Human factors engineering, etc. Section 6.2 Resilience: Consideration of rebalancing issues in the event of disruptions	#0, #2, #3, #5, #9, #10

**Table 4:** C-dimensional related literature.

References	Analytical Dimension			Research Objectives	Solution Approach
	Layout	Production	Uncertainty		
[22]	√			Optimizing U-Shaped Production Line Balancing Problem	integer programming model, use Lingo to solve for the minimum production cycle.
[23]	√			DLBP with Multiple Solution Space	Multi-objective mathematical model, ring topology pollination algorithm (RTFPA)
[24]		√		Design & implementation of the production line in garment industry	Quantitative research methods, lean manufacturing tools, 5S
[25]	√			SALBP	Variable depth local search algorithm, heuristic algorithm
[26]	√			Assembly line optimization and balancing	GAB and genetic transfer learning (GTL) methods
[27]	√			Clothing production line balancing optimization	Improvements in genetic algorithms and computer simulation technology
[28]		√		Balancing The Shirt Production Line	Integer programming model considering dual constraints of manpower and machinery, ranking position weighting method
[29]		√		balancing U-Shaped disassembly line with flexible workstations and spatial constraints	Hybrid integer nonlinear programming model and constraint programming model, hybrid constraint programming and cross-entropy approach
[30]	√			Automobile assembly line balancing	GA, decision support systems
[31]	√			Load balancing of dual-side assembly lines	Mathematical programming models, deep reinforcement learning algorithms
[32]	√			Efficiently balancing assembly lines	Heuristic algorithms, multi-feature optimization models
[33]		√		Customized product line balancing	Two-step process method, component grouping, task and worker allocation optimization model
[34]		√		Cable production line balancing issues	Rank positional weight method, heuristic method, workstation load balancing
[35]		√		Production cycle time and balance rate	Non-dominated Sorting Genetic Algorithm II(NSGA-II)
[36]		√		Uneven workload among workers	Dual-objective integer nonlinear programming model,
[37]		√		Mixed Production Line Optimization of Industrialized Building	combining NSGA-II with multi-objective simulated annealing meta-heuristic method
[38]		√		Balancing hybrid assembly lines in multi-demand scenarios	Genetic algorithms, sequence optimization, and buffer allocation for evaluating individual fitness functions
[39]		√		Optimizing remanufacturing cycle time and overall balance rate (CBR)	Production rhythm optimization mathematical model, particle swarm optimization algorithm
[40]			√	Balancing production lines with uncertain demand	Mixed-integer linear programming model, improved migratory bird optimization algorithm
[41]			√	Balancing mixed-flow assembly lines in uncertain environments	Interval Type-2 Fuzzy Set Theory
[42]			√	balancing and sequencing problems of flexible mixed model assembly lines	AND/OR graph modeling, iterative decomposition methods
[43]		√		Efficiency of mixed assembly lines	Ant colony optimization algorithm, production line scheduling optimization
[44]			√	The multi-manned joint assembly line balancing	heuristic algorithm based on adaptive large neighborhood search framework

**Table 5:** H-dimensional related literature.

References	Analytical Dimension			Research Objectives	Solution Approach
	Product Sequencing	Worker Assignment	Material Handling		
[64]		√		Optimization of disassembly line balancing considering worker skill differences	Mixed-integer programming (MIP) model, Based on incentive strategy NSGA-II
[65]		√		Balancing human-machine collaboration assembly lines considering ergonomic risks	Multi-objective optimization mathematical model, improved multi-objective particle swarm optimization algorithm
[66]		√		Automated allocation of production line tasks	Designing decision support systems for interactive and iterative workflows
[67]		√		Assembly line design and load balancing under parallel task conditions	Mixed integer programming model, simulated annealing algorithm with improved strategy
[68]		√		Assembly line balancing and worker allocation	Allocation strategy for worker performance variability, dual-objective linear programming model
[69]		√		Clothing production line balancing	Task modularization, dual allocation of tasks and workers
[70]			√	Optimizing the Material-Product Transformation Processes	string diagram, Minimization of resource movement, analysis of production activities, layout design
[71]			√	Simulation of in-house logistics operations for manufacturing	Building a logistics simulation model for an automobile manufacturing factory
[72]	√			Balance optimization of mixed-flow assembly lines under random sequences	Branch-and-bound algorithm, exact methods, heuristic extension schemes

**Table 6:** V-Dimension Related Literature.

References	Analytical Dimension		Research Objectives	Solution Approach
	Upstream	Downstream		
[93]	√		Consider the impact of ergonomic factors on production line efficiency during the design phase	Designing models that maximize production line efficiency, Linearization solution
[94]	√		Production line balancing during the design phase	Process Planning Forecasting Analysis Method
[95]	√		Production Efficiency of Mixed Flow Assembly Lines for Wall Components	A hybrid approach combining configuration modeling and discrete event simulation techniques
[96]	√		Robot assembly line balancing	Process time distribution simulation, Evaluating the impact of different process time distributions
[97]	√		Incorporating car-sequencing rules in the planning of mixed-model assembly lines	Design genetic algorithms combine balancing problems with semi-random production sequences
[98]		√	Mobile phone assembly line production process combination and workstation division	Dual production line mixing workshop, mixing workshop optimization model, heuristic algorithm
[99]		√	Research on Production Costs and Process Optimization	Measure workstation time consumption, balance workstation method
[100]		√	Optimize production processes, Reduce supply chain costs	Value stream mapping, line balancing method, ECRS
[101]		√	Production line fluctuation issues	Improving mathematical models, Segment work-in-process inventory
[102]		√	Waste of idle resources at production sites, Low production line balance rate	Artificial Intelligence-based Data Mining Intelligent Manufacturing Management System

**Table 7:** E-Dimension Related Literature.

References	Analytical Dimension		Research Objectives	Solution Approach
	Sustainability	Resilience		
[106]	√		U-shaped disassembly line balancing problem	Improved Fuzzy Multi-Objective Particle Swarm Optimization Algorithm (FMOPSO), interval Type-2 trapezoidal fuzzy set (IT2TFS)
[107]	√		Remanufacturing dismantling line balancing	Random parallel disassembly line balancing model, high-order heuristic algorithm (HH) for simulated annealing
[108]		√	preventive maintenance integrated disassembly line balancing	Mixed integer programming model, Deep-Q-network-enhanced aquila-equilibrium hyper-heuristic algorithm
[109]		√	Minimizing task reallocation in multi-product reconfigurable production lines	Mixed-integer linear programming (MILP) model, MILP-based heuristic algorithm
[110]		√	Reconfigurable production line balancing, energy consumption minimization	Time-indexed integer linear programming model, heuristic algorithm
[111]	√		RMS balancing and planning	Double-layer optimization model, discrete whale optimization algorithm
[112]	√		Collaborative robot assembly line optimization	MILP model, neighborhood search simulated annealing algorithm (SA)
[113]		√	Intelligent adaptive production line rebalancing and maintenance	Multi-agent reinforcement learning (MARL)
[114]		√	Dynamic Scheduling and Reconfiguration of Distributed Reconfigurable Production Lines	Heuristic dynamic rescheduling method, iterated greedy algorithm

## Data Availability

Not applicable.

## References

[1] Tang S.-M., Pei W., Chuang M.-H. (2025). PV chart management innovation based on production balance ratio: A case study of Company S’s GaAs process improvement for compound semiconductors. Int. J. Adv. Manuf. Technol..

[2] Teshome M.M., Meles T.Y., Yang C.-L. (2024). Productivity improvement through assembly line balancing by using simulation modeling in case of Abay garment industry Gondar. Heliyon.

[3] Chao B., Zhang C., Zhang Y., Guo H., Ren Y., Zhang H. (2022). Research on optimization and simulation of sand casting production line based on VSM. Int. J. Lean Six Sigma.

[4] Feng L. (2022). Research on Intelligent Production Line Design and Dynamic Balance for 3C Products. Wirel. Commun. Mob. Comput..

[5] Dimeny I., Koltai T. (2024). Comparison of MILP and CP models for balancing partially automated assembly lines. Cent. Eur. J. Oper. Res..

[6] Breznik M., Buchmeister B., Herzog N.V. (2023). Assembly Line Optimization Using MTM Time Standard and Simulation Modeling-A Case Study. Appl. Sci..

[7] Kadarova J., Janekova J., Suhanyiova A. (2022). Possibilities to Increase Assembly Line Productivity Using Different Management Approaches. Processes.

[8] Laroca A., Pereira M.T., Silva F.J.G., Oliveira M.J.G.P. (2024). Optimization of an Air Conditioning Pipes Production Line for the Automotive Industry-A Case Study. Systems.

[9] Battaia O., Dolgui A., Guschinsky N. (2024). An exact method for machining lines design with equipment selection and line balancing. Int. J. Prod. Res..

[10] Li S., Butterfield J., Murphy A. (2023). A New Multi-Objective Genetic Algorithm for Assembly Line Balancing. J. Comput. Inf. Sci. Eng..

[11] Telemeci Y.E., Azizoglu M. (2024). Type-II transfer line Balancing problem—A branch and bound approach. Comput. Ind. Eng..

[12] Ghandi S., Masehian E. (2025). An efficient solution to the simple assembly line balancing problem type 1 using iterated local search. Eng. Appl. Artif. Intell..

[13] Deliktas D., Aydin D. (2023). An artificial bee colony based-hyper heuristic algorithm with local search for the assembly line balancing problems. Eng. Comput..

[14] Moncayo-Martinez L.A., He N., Arias-Nava E.H. (2025). Minimising by Simulation-Based Optimisation the Cycle Time for the Line Balancing Problem in Real-World Environments. Appl. Stoch. Models Bus. Ind..

[15] Nourmohammadi A., Fathi M., Ng A.H.C. (2024). Balancing and scheduling human-robot collaborated assembly lines with layout and objective consideration. Comput. Ind. Eng..

[16] Battaïa O., Dolgui A. (2022). Hybridizations in line balancing problems: A comprehensive review on new trends and formulations. Int. J. Prod. Econ..

[17] Naldi L.D., Galizia F.G., Bortolini M. (2025). Balancing storage cost and customization time in product platform design: A bi-objective optimization model. Int. J. Adv. Manuf. Technol..

[18] Torkul O., Selvi I.H., Şişci M., Öge M. (2024). An integrated simulation-data envelopment analysis approach for impact of line-seru conversion. RAIRO—Oper. Res..

[19] Jiao Y., Cao N., Li J., Li L., Deng X. (2022). Balancing a U-Shaped Assembly Line with a Heuristic Algorithm Based on a Comprehensive Rank Value. Sustainability.

[20] Yelles-Chaouche A.R., Gurevsky E., Brahimi N., Dolgui A. (2024). Optimizing modular equipment in the design of multi-product reconfigurable production lines. Comput. Ind. Eng..

[21] Zhang Z., Tang Q., Chica M., Li Z. (2023). Reinforcement Learning-Based Multiobjective Evolutionary Algorithm for Mixed-Model Multimanned Assembly Line Balancing Under Uncertain Demand. IEEE Trans. Cybern..

[22] Kuo Y., Yang T., Huang T.-L. (2022). Optimizing U-Shaped Production Line Balancing Problem with Exchangeable Task Locations and Walking Times. Appl. Sci..

[23] Zhang L., Jin R., Geng X., Hu J., Bao H. (2025). Flower Pollination Algorithm with Ring Topology for Multisolution Spaces to Solve the Disassembly Line Balancing Problem. J. Manuf. Sci. Eng.-Trans. ASME.

[24] Kumar D.V., Mohan G.M., Mohanasundaram K.M. (2022). Design & implementation of the production line in garment industry. Ind. Textila.

[25] Álvarez-Miranda E., Pereira J., Vargas C., Vilà M. (2023). Variable-depth local search heuristic for assembly line balancing problems. Int. J. Prod. Res..

[26] Gao H., Zhang Y., Bi Z. (2023). Genetic Transfer Learning for Optimizing and Balancing of Assembly Lines. IEEE Trans. Ind. Inform..

[27] Jiang L., Duan J.J., Zheng R.P., Shen H.N., Li H., Xu J. (2023). Optimization and Simulation of Garment Production Line Balance Based on Improved GA. Int. J. Simul. Model..

[28] Yildiz S.T., Karabay G. (2022). Balancing The Shirt Production Line Under Different Operational Constraints Using An Integer Programming Model. Tekst. Konfeksiyon.

[29] Zhang Y., Zhang Z., Chu F., Mammar S. (2025). A hybrid constraint programming and cross-entropy approach for balancing U-Shaped disassembly line with flexible workstations and spatial constraints. J. Ind. Inf. Integr..

[30] Didden J.B.H.C., Lefeber E., Adan I.J.B.F., Panhuijzen I.W.F. (2023). Genetic algorithm and decision support for assembly line balancing in the automotive industry. Int. J. Prod. Res..

[31] Jia G., Zhang Y., Shen S., Liu B., Hu X., Wu C. (2023). Load Balancing of Two-Sided Assembly Line Based on Deep Reinforcement Learning. Appl. Sci..

[32] Alhomaidi E., Askin R.G. (2024). Exact and approximation heuristic of mixed model assembly line balancing with parallel lines and task-dependent tooling consideration. Comput. Ind. Eng..

[33] Pilati F., Lelli G., Regattieri A. (2022). Assembly line balancing and activity scheduling for customised products manufacturing. Int. J. Adv. Manuf. Technol..

[34] Celik M.T., Arslankaya S. (2023). Solution of the assembly line balancing problem using the rank positional weight method and Kilbridge and Wester heuristics method: An application in the cable industry. J. Eng. Res..

[35] Zhang Q., Li H., Shen S., Cao W., Jiang J., Tang W., Hu Y. (2024). Multi-objective optimization of the mixed-flow intelligent production line for automotive MEMS pressure sensors. Appl. Intell..

[36] Wu Y., Zeng S., Li B., Yu Y. (2024). A bi-objective approach for the multi-skilled worker assignment of a hybrid assembly line-seru production system. RAIRO—Oper. Res..

[37] Chen X., Yu F., Zhou H., Li Z., Wu K.-J., Qian X. (2022). Mixed Production Line Optimization of Industrialized Building Based on Ant Colony Optimization Algorithm. Adv. Civ. Eng..

[38] Wang K., Han Q., Li Z. (2023). Mixed-model assembly line balancing problem in multi-demand scenarios. Int. J. Ind. Eng. Comput..

[39] Liu Q., Wei X., Wang Q., Song J., Lv J., Liu Y., Tang O. (2024). An investigation of mixed-model assembly line balancing problem with uncertain assembly time in remanufacturing. Comput. Ind. Eng..

[40] Shao H., Moroni G., Li A., Xu L. (2022). Heuristic Approach for a Combined Transfer Line Balancing and Buffer Allocation Problem Considering Uncertain Demand. Appl. Sci..

[41] Zhang X., Fathollahi-Fard A.M., Tian G., Yaseen Z.M., Pham D.T., Zhao Q., Wu J. (2024). Human-Robot Collaboration in Mixed-Flow Assembly Line Balancing under Uncertainty: An Efficient Discrete Bees Algorithm. J. Ind. Inf. Integr..

[42] Peng Y., Zhang L., Xia B., Han Y. (2023). Research on balancing and sequencing problems of flexible mixed model assembly lines with alternative precedence relations. Int. J. Prod. Res..

[43] Tiacci L. (2024). Combining balancing, sequencing and buffer allocation decisions to improve the efficiency of mixed-model asynchronous assembly lines. Comput. Ind. Eng..

[44] Zangaro F., Minner S., Battini D. (2023). The multi-manned joint assembly line balancing and feeding problem. Int. J. Prod. Res..

[45] Boysen N., Schulze P., Scholl A. (2022). Assembly line balancing: What happened in the last fifteen years?. Eur. J. Oper. Res..

[46] Jiao Y., Su X., Li L., Wu Z. (2024). An improved ant colony optimization algorithm for two-sided U-type assembly line balancing problems. Eng. Optim..

[47] Wang K., Li Y., Guo J., Gao L., Li X. (2024). Dynamic Balancing of U-Shaped Robotic Disassembly Lines Using an Effective Deep Reinforcement Learning Approach. IEEE Trans. Ind. Inform..

[48] Demiralay Y.D., Kara Y. (2024). Profit-oriented balancing of two-sided disassembly lines with resource-dependent task times. Robot. Intell. Autom..

[49] Liao S.-G., Zhang Y.-B., Sang C.-Y., Liu H. (2023). A genetic algorithm for balancing and sequencing of mixed-model two-sided assembly line with unpaced synchronous transfer. Appl. Soft Comput..

[50] Zhao L., Tang Q., Zhang Z., Zhu Y. (2024). A Knowledge-Assisted Variable Neighborhood Search for Two-Sided Assembly Line Balancing Considering Preventive Maintenance Scenarios. IEEE Trans. Syst. Man Cybern. Syst..

[51] Zhang Y., Zhang Z., Yin T., Liang W. (2022). Mathematical formulation and an improved moth–flame optimization algorithm for parallel two-sided disassembly line balancing based on fixed common stations. J. Comput. Des. Eng..

[52] Mao Z., Zhang J., Sun Y., Fang K., Huang D. (2025). Balancing parallel assembly lines with human-robot collaboration: Problem definition, mathematical model and tabu search approach. Int. J. Prod. Res..

[53] Guo X., Zhou L., Zhang Z., Qi L., Wang J., Qin S., Cao J. (2024). Multi-Objective Optimization of Multi-Product Parallel Disassembly Line Balancing Problem Considering Multi-Skilled Workers Using a Discrete Chemical Reaction Optimization Algorithm. Comput. Mater. Contin..

[54] Huo J., Lee C.K.M. (2024). A Benders’ Decomposition Algorithm for Balancing and Sequencing of the Mixed-Model Multi-Manned Assembly Lines. IEEE Trans. Syst. Man Cybern.-Syst..

[55] Yuan G., Liu X., Zhu C., Wang C., Zhu M., Sun Y. (2023). Multi-objective coupling optimization of electrical cable intelligent production line driven by digital twin. Robot. Comput. Manuf..

[56] Sun X., Guo S., Guo J., Du B., Yang Z., Wang K. (2024). A Pareto-based hybrid genetic simulated annealing algorithm for multi-objective hybrid production line balancing problem considering disassembly and assembly. Int. J. Prod. Res..

[57] Tao R., Tao L., Su B., Javanmardi E. (2024). Neighbourhood-search-enhanced non-dominated sorting genetic algorithm-III for multi-objective assembly line balancing problem considering operator skill levels and carbon footprint. Eng. Optim..

[58] Meng K., Tang Q., Zhang Z. (2023). Balancing and sequencing of mixed-model assembly line considering preventive maintenance scenarios: Mathematical model and a migrating birds optimization algorithm. Flex. Serv. Manuf. J..

[59] Dang Z., Xie N. (2024). Assembly line balancing and capacity evaluation based on interval grey processing time. Grey Syst.-Theory Appl..

[60] Li Z., Sikora C.G.S., Kucukkoc I. (2024). Chance-constrained stochastic assembly line balancing with branch, bound and remember algorithm. Ann. Oper. Res..

[61] Sahin M.C., Tural M.K. (2023). Robotic stochastic assembly line balancing. Flex. Serv. Manuf. J..

[62] Aslan S. (2023). Mathematical model and a variable neighborhood search algorithm for mixed-model robotic two-sided assembly line balancing problems with sequence-dependent setup times. Optim. Eng..

[63] Feng L., Wang Y., Fang X., Yu H., Zhang S. (2024). Two-sided resource-constrained assembly line balancing problem: A new mathematical model and an improved genetic algorithm. Swarm Evol. Comput..

[64] Yin T., Zhang Z., Wu T., Zeng Y., Zhang Y., Liu J. (2022). Multimanned partial disassembly line balancing optimization considering end-of-life states of products and skill differences of workers. J. Manuf. Syst..

[65] Yin X., Yang Y., Li X., Wu R., Guo A., Zhao Q. (2025). Research on the balancing problem of human–robot collaborative assembly line in SMEs considering ergonomic risk and cost. Comput. Ind. Eng..

[66] Fink C., Bodin U., Schelen O. (2025). Why decision support systems are needed for addressing the theory-practice gap in assembly line balancing. J. Manuf. Syst..

[67] Wang K., Zhang Y., Li Z. (2025). Research on workload balance problem of mixed model assembly line under parallel task strategy. Int. J. Ind. Eng. Comput..

[68] Katiraee N., Calzavara M., Finco S., Battaïa O., Battini D. (2023). Assembly line balancing and worker assignment considering workers’ expertise and perceived physical effort. Int. J. Prod. Res..

[69] Kim M., Kim S. (2023). Effects of step-by-step line balancing in apparel assembly line. J. Eng. Fibers Fabr..

[70] Kucuk M., Isler M., Guner M. (2022). Optimizing the Material-Product Transformation Processes in the Clothing Manufacturing Line. Tekst. Konfeksiyon.

[71] Coelho F., Macedo R., Relvas S., Barbosa-Póvoa A. (2022). Simulation of in-house logistics operations for manufacturing. Int. J. Comput. Integr. Manuf..

[72] Sikora C.G.S. (2024). Balancing mixed-model assembly lines for random sequences. Eur. J. Oper. Res..

[73] Meng K., Li S., Han Z. (2025). Optimizing mixed-model assembly line efficiency under uncertain demand: A Q-Learning-Inspired differential evolution algorithm. Comput. Ind. Eng..

[74] Zhang Z., Zhu L., Chen Y., Guan C. (2022). A multi-objective hybrid evolutionary search algorithm for parallel production line balancing problem including disassembly and assembly tasks. Int. Trans. Oper. Res..

[75] Elyasi M., Selcuk Y.S., Özener O.Ö., Coban E. (2024). Imperialist competitive algorithm for unrelated parallel machine scheduling with sequence-and-machine-dependent setups and compatibility and workload constraints. Comput. Ind. Eng..

[76] Peng F., Zheng L. (2023). Integrating real-time manufacturing data into a novel serial two-stage adaptive alternate genetic fireworks algorithm for solving stochastic type-II simple assembly line balancing problem. Complex Intell. Syst..

[77] Chourabi Z., Khedher F., Babay A., Cheikhrouhou M. (2024). Developing a metaheuristic model for the general assembly line balancing optimization based on a new workforce performance index: A case study in the garment industry. J. Text. Inst..

[78] Kim G.-Y., Yun J., Lee C., Lim J., Kim Y., Noh S.D. (2024). Data-driven analysis and human-centric assignment for manual assembly production lines. Comput. Ind. Eng..

[79] Li Y.C., Wang X. (2024). Human-robot collaboration assembly line balancing considering cross-station tasks and the carbon emissions. Adv. Prod. Eng. Manag..

[80] Perez-Wheelock R.M., Ou W., Yenradee P., Huynh V.-N. (2022). A Demand-Driven Model for Reallocating Workers in Assembly Lines. IEEE Access.

[81] Alhomaidhi E. (2024). Enhancing efficiency and adaptability in mixed model line balancing through the fusion of learning effects and worker prerequisites. Int. J. Ind. Eng. Comput..

[82] Abdous M.-A., Delorme X., Battini D., Sgarbossa F. (2025). Scenario-based optimization and simulation framework for human-centered Assembly Line Balancing. Int. J. Prod. Econ..

[83] Kulac S., Kiraz A. (2024). An integrated ergonomic risk assessment framework based on fuzzy logic and IVSF-AHP for optimising ergonomic risks in a mixed-model assembly line. Ergonomics.

[84] Tiacci L. (2023). Assigning rest times to workers in assembly lines with ergonomically hazardous tasks: An approach to defend companies’ profitability. Int. J. Prod. Res..

[85] Noda K., Sun J., Yamamoto H., Dou R.-L. (2025). A study of optimal assignment model considering quality and worker level in limited-cycle with multiple periods for smart manufacturing. Int. J. Ind. Eng.-Theory Appl. Pract..

[86] Kang H.-Y., Lee A.H.I., Su Y.-X. (2025). Multi-objective mixed-model assembly line balancing with hierarchical worker assignment: A case study of gear reducer manufacturing operations. Int. J. Ind. Eng. Comput..

[87] Zeng F., Fan C., Shirafuji S., Wang Y., Nishio M., Ota J. (2025). Task allocation and scheduling to enhance human–robot collaboration in production line by synergizing efficiency and fatigue. J. Manuf. Syst..

[88] Abdous M.A., Delorme X., Battini D., Sgarbossa F., Berger-Douce S. (2023). Assembly line balancing problem with ergonomics: A new fatigue and recovery model. Int. J. Prod. Res..

[89] Mumtaz J., Minhas K.A., Rauf M., Yue L., Chen Y. (2024). Solving line balancing and AGV scheduling problems for intelligent decisions using a Genetic-Artificial bee colony algorithm. Comput. Ind. Eng..

[90] Arik O.A., Yufka P.N. (2025). A new mathematical model approach with assembly line feeding based Milk-Run system. J. Fac. Eng. Archit. Gazi Univ..

[91] Hou W., Zhang S. (2024). Assembly line balancing and optimal scheduling for flexible manufacturing workshop. J. Mech. Sci. Technol..

[92] Feng J., Che A. (2023). A note on integrated disassembly line balancing and routing problem. Int. J. Prod. Res..

[93] Alfaro-Pozo R., Bautista-Valhondo J. (2024). Impact of limiting the ergonomic risk on the economic and productive efficiency of an assembly line. Int. J. Prod. Res..

[94] Aicha M., Belhadj I., Hammadi M. (2023). Disassembly Process Planning and Its Lines Balancing Prediction. Int. J. Precis. Eng. Manuf.-Green Technol..

[95] Grenzfurtner W., Pichler E., Gronalt M. (2024). Increasing the output of mixed-model assembly lines for industrialised housebuilding: Learnings from a case-based simulation study. Int. J. Prod. Res..

[96] Stade D., Spoor J.M., Manns M., Ovtcharova J. (2024). Process time distribution simulation in robotic assembly line balancing. Int. J. Prod. Res..

[97] Sikora C.G.S., Tiacci L. (2025). Incorporating car-sequencing rules in the planning of mixed-model assembly lines. Int. J. Prod. Res..

[98] Zhao R., Zou G., Su Q., Zou S., Deng W., Yu A., Zhang H. (2022). Digital Twins-Based Production Line Design and Simulation Optimization of Large-Scale Mobile Phone Assembly Workshop. Machines.

[99] Kádárová J., Kočišová M., Teplická K., Suhányiová A., Lachvajderová L. (2022). Optimization of Costs and Production Process of Fire Dampers. Appl. Sci..

[100] Kittichotsatsawat Y., Wattanutchariya W., Jongjareonrak A., Seesuriyachan P. (2025). Enhancing Manufacturing Operations Within the Supply Chain for Sustainable Frozen Shrimp Production. Sustainability.

[101] Kampa A., Paprocka I. (2024). The Influence of the Assembly Line Configuration and Reliability Parameter Symmetry on the Key Performance Indicators. Symmetry.

[102] Guo Y., Zhang W., Qin Q. (2023). Intelligent manufacturing management system based on data mining in artificial intelligence energy-saving resources. Soft Comput..

[103] Hu J., Zhang Z., Qiu H., Zhao J., Xu X. (2022). Enhanced Hybrid Ant Colony Optimization for Machining Line Balancing Problem with Compound and Complex Constraints. Appl. Sci..

[104] Liu J., Lv Y. (2023). A Multi-Object Genetic Algorithm for the Assembly Line Balance Optimization in Garment Flexible Job Shop Scheduling. Intell. Autom. Soft Comput..

[105] He J., Chu F., Dolgui A., Anjos M. (2024). Multi-objective disassembly line balancing and related supply chain management problems under uncertainty: Review and future trends. Int. J. Prod. Econ..

[106] Zhang H., Huang Z., Wang D., Tian G., Wang W. (2025). U-shaped disassembly line balancing problem under interval Type-2 trapezoidal fuzzy set: Modeling and solution method. Eng. Appl. Artif. Intell..

[107] Hu Y., Liu C., Zhang M., Jia Y., Xu Y. (2023). A Novel Simulated Annealing-Based Hyper-Heuristic Algorithm for Stochastic Parallel Disassembly Line Balancing in Smart Remanufacturing. Sensors.

[108] Huang Y., Zhou B. (2025). Deep-Q-network-enhanced aquila-equilibrium hyper-heuristic algorithm for preventive maintenance integrated disassembly line balancing involving worker redeployment. Comput. Ind. Eng..

[109] Yelles-Chaouche A.R., Gurevsky E., Brahimi N., Dolgui A. (2022). Minimizing task reassignments under balancing multi-product reconfigurable manufacturing lines. Comput. Ind. Eng..

[110] Delorme X., Gianessi P. (2024). Line balancing and task scheduling to minimise power peak of reconfigurable manufacturing systems. Int. J. Prod. Res..

[111] Delorme X., Cerqueus A., Gianessi P., Lamy D. (2023). RMS balancing and planning under uncertain demand and energy cost considerations. Int. J. Prod. Econ..

[112] Nourmohammadi A., Fathi M., Ng A.H.C. (2022). Balancing and scheduling assembly lines with human-robot collaboration tasks. Comput. Oper. Res..

[113] Nikkerdar M., ElMaraghy W. (2025). Smart adaptable assembly line rebalancing and maintenance. Int. J. Adv. Manuf. Technol..

[114] Yang S., Wang J., Li W., Xu Z. (2025). Integrated optimisation of dynamic scheduling and reconfiguration for distributed reconfigurable flowshops via iterated greedy algorithm. Int. J. Syst. Sci.-Oper. Logist..

[115] Guo L., Zhang Z., Wu T., Zhang Y., Zeng Y., Xie X. (2024). Green and efficient-oriented human-robot hybrid partial destructive disassembly line balancing problem from non-disassemblability of components and noise pollution. Robot. Comput. Manuf..

[116] Tian G., Zhang C., Zhang X., Feng Y., Yuan G., Peng T., Pham D.T. (2023). Multi-Objective Evolutionary Algorithm with Machine Learning and Local Search for an Energy- Efficient Disassembly Line Balancing Problem in Remanufacturing. J. Manuf. Sci. Eng.-Trans. ASME.

[117] Sun B., Liu J., Li G. (2025). Joint balancing and sequencing optimization for type-II robotic mixed-model assembly line considering energy consumption. Ann. Oper. Res..

[118] Elmolouk R.S., El-Kharbotly A.M.K., Taha R.B. (2025). Optimization of time and energy in straight one-sided robotic assembly lines. Sci. Rep..

[119] Chi Y., Qiao Z., Li Y., Li M., Zou Y. (2022). Type-1 Robotic Assembly Line Balancing Problem That Considers Energy Consumption and Cross-Station Design. Systems.

[120] Tian G., Zhang C., Fathollahi-Fard A.M., Li Z., Zhang C., Jiang Z. (2022). An Enhanced Social Engineering Optimizer for Solving an Energy-Efficient Disassembly Line Balancing Problem Based on Bucket Brigades and Cloud Theory. IEEE Trans. Ind. Inform..

[121] Shen Y., Lu W., Sheng H., Liu Y., Tian G., Zhang H., Li Z. (2024). Human-Robot Collaboration on a Disassembly-Line Balancing Problem with an Advanced Multiobjective Discrete Bees Algorithm. Symmetry.

[122] Guo X., Chen L., Qi L., Wang J., Qin S., Chatterjee M., Kang Q. (2025). Multifactory Disassembly Process Optimization Considering Worker Posture. IEEE Trans. Comput. Soc. Syst..

[123] Wei T., Guo X., Zhou M., Wang J., Liu S., Qin S., Tang Y. (2025). A Multiobjective Discrete Harmony Search Optimizer for Disassembly Line Balancing Problems Considering Human Factors. IEEE Trans. Human-Mach. Syst..

[124] Qi L., Zeng Q., Liu S., Wang J., Qin S., Guo X. (2025). Twin Delayed Deep Deterministic Policy Gradient Algorithm for a Heterogeneous Multifactory Remanufacturing Optimization Problem. IEEE Trans. Comput. Soc. Syst..

[125] Cui X., Meng Q., Wang J., Guo X., Liu P., Qi L., Qin S., Ji Y., Hu B. (2025). An Evolutionary Learning Whale Optimization Algorithm for Disassembly and Assembly Hybrid Line Balancing Problems. Mathematics.

[126] Zacharia P.T., Xidias E.K., Nearchou A.C. (2024). The fuzzy human-robot collaboration assembly line balancing problem. Comput. Ind. Eng..

[127] Kheirabadi M., Keivanpour S., Chinniah Y.A., Frayret J.-M. (2023). Human-robot collaboration in assembly line balancing problems: Review and research gaps. Comput. Ind. Eng..

[128] Shao Z., Li W., Tan Y., Otto K. (2024). A systematic energy-aware scheduling framework for manufacturing factories integrated with renewables. Int. J. Prod. Res..

[129] Le T.T., Ferraris A., Dhar B.K. (2023). The contribution of circular economy practices on the resilience of production systems: Eco-innovation and cleaner production’s mediation role for sustainable development. J. Clean. Prod..

[130] Albus M., Hornek T., Kraus W. (2025). Towards scalability for resource reconfiguration in robotic assembly line balancing problems using a modified genetic algorithm. J. Intell. Manuf..

[131] Albus M., Huber M.F. (2023). Resource reconfiguration and optimization in brownfield constrained Robotic Assembly Line Balancing Problems. J. Manuf. Syst..

[132] Khan A.S., Khan R., Saleem W., Salah B., Alkhatib S. (2022). Modeling and Optimization of Assembly Line Balancing Type 2 and E (SLBP-2E) for a Reconfigurable Manufacturing System. Processes.

[133] Tang Q., Meng K., Cheng L., Zhang Z. (2022). An improved multi-objective multifactorial evolutionary algorithm for assembly line balancing problem considering regular production and preventive maintenance scenarios. Swarm Evol. Comput..

[134] Wang H.-K., Chou C.-W., Wang C.-H., Ho L.-A. (2024). Sustainable scheduling of TFT-LCD cell production: A hybrid dispatching rule and two-phase genetic algorithm. Int. J. Prod. Econ..

[135] Beldar P., Fathi M., Nourmohammadi A., Delorme X., Battaïa O., Dolgui A. (2025). Transfer line balancing problem: A comprehensive review, classification, and research avenues. Comput. Ind. Eng..

[136] Schlueter M.J., Ostermeier F.F. (2022). Dynamic line balancing in unpaced mixed-model assembly lines: A problem classification. Cirp J. Manuf. Sci. Technol..

[137] Li Y., Saldanha-Da-Gama F., Liu M., Yang Z. (2023). A risk-averse two-stage stochastic programming model for a joint multi-item capacitated line balancing and lot-sizing problem. Eur. J. Oper. Res..

[138] Schäfer L., Tse S., May M.C., Lanza G. (2024). Assisted production system planning by means of complex robotic assembly line balancing. J. Manuf. Syst..

[139] Li Y., Qiao Z., Chi Y., Guo L., Yan R. (2024). Robotic assembly line balancing considering the carbon footprint objective with cross-station design. Comput. Ind. Eng..

[140] Mumcu Y.K. (2024). Solution approach using heuristic and artificial neural networks methods in assembly line balancing problems: A case study in the lighting industry. Heliyon.

[141] Miao Q., Bai Z., Liu X., Awais M. (2023). Modelling and numerical analysis for seru system balancing with lot splitting. Int. J. Prod. Res..

[142] Paprocka I., Skolud B. (2022). A Predictive Approach for Disassembly Line Balancing Problems. Sensors.

[143] Wang X., Lin C., Yang S., Chen J., Liu B., Chipusu K. (2024). A deep learning approach for balance optimisation of patch panel assembly line. J. Eng. Des..

[144] Wang R., Xin T., Jia S., Ren D., Li M. (2024). Production line balance problem identification and improvement based on decision tree: A case study of commercial air conditioner production line. Sci. Prog..

[145] Zeng Y., Zhang Z., Zhang Y., Liang W., Song H. (2025). Modelling and optimization of line efficiency for preventive maintenance of robot disassembly line. J. Manuf. Syst..

[146] Kim M., Kim S. (2024). Development of a dedicated process simulator for the digital twin in apparel manufacturing: A case study. Int. J. Cloth. Sci. Technol..

[147] Tarek N., Algarni A.D., El-Hefnawy N.A., Abdel-Kader H., Abdelatey A. (2025). Knowledge Graph-Enhanced Digital Twin Framework for Optimized Job Shop Scheduling in Smart Manufacturing. IEEE Access.

[148] Chawla S., Singari R.M. (2024). Analysing the Bottleneck in Crankcase Cover Manufacturing using Simulation and Modelling. J. Sci. Ind. Res..

[149] Yin T., Zhang Z., Liang W., Zeng Y., Zhang Y. (2023). Multi-Man–Robot Disassembly Line Balancing Optimization by Mixed-Integer Programming and Problem-Oriented Group Evolutionary Algorithm. IEEE Trans. Syst. Man Cybern. Syst..

[150] Jin L., Zhang X., Fang Y., Pham D.T. (2022). Transfer Learning-Assisted Evolutionary Dynamic Optimisation for Dynamic Human-Robot Collaborative Disassembly Line Balancing. Appl. Sci..

[151] Cai J., Xue H., Zheng C., Shi H. (2025). Improved dual-population genetic algorithm to solve human-robot collaborative assembly line balancing problem. Int. J. Prod. Res..

[152] Fathi M., Sepehri A., Ghobakhloo M., Iranmanesh M., Tseng M.-L. (2024). Balancing assembly lines with industrial and collaborative robots: Current trends and future research directions. Comput. Ind. Eng..

[153] Yin T., Wu J., Cao J., Huang Y., Li C., Long J. (2025). Multi-man–robot collaborative disassembly line balancing optimization via mixed-integer programming and genetic Jaya algorithm. J. Clean. Prod..

